# Bone Scintigraphy for Guidance of Targeted Treatment of Vertebral Compression Fractures

**DOI:** 10.3390/jcm13123627

**Published:** 2024-06-20

**Authors:** Elite Arnon-Sheleg, Daniel Weiner, Saeda Haj, Alon Rod, Nimrod Rahamimov

**Affiliations:** 1 Department of Radiology and Nuclear Medicine, Galilee Medical Center, Nahariya 2210001, Israel; 2 Azrieli Faculty of Medicine, Bar-Ilan University, Safed 5290002, Israel; 3 Department of Orthopedics B and Spine Surgery, Galilee Medical Center, Nahariya 2210001, Israelalonr@gmc.gov.il (A.R.)

**Keywords:** bone scintigraphy, computed tomography, vertebral compression fracture, occult fracture, osteoporosis

## Abstract

**Background:** Vertebral compression fractures (VCFs) are prevalent in the elderly population and might be the source of back pain if they are fresh and yet unhealed. In many cases, it is a diagnostic challenge to differentiate fresh VCFs from healed united fractures, which retain similar radiographic characteristics but no longer generate pain. This information is crucial for appropriate management. The aim of this study was to evaluate the role of bone scintigraphy (BS) in identifying fresh VCFs appropriate for targeted treatment when compared to the findings of Computerized Tomography (CT). **Methods**: We retrospectively reviewed 190 patients with back pain suspected to stem from a recent VCF that underwent both a CT and a BS and compared the imaging patterns per vertebra. **Results**: The studies were concordant in the majority of cases (95.5%), diagnosing 84.4% normal vertebrae, 6.4% acute VCFs, and 4.7% chronic VCFs. However, in 37 patients, 45 occult acute VCFs were only detected on BS and not on CT. Multivariate logistic regression analysis revealed that these patients were older and had lower bone density compared to the rest of the study population. Additionally, 40 patients had acute VCFs visible on CT, but with no increased or low intensity uptake on BS. These cases were associated with a shorter time period between trauma and BS, a higher prevalence of male patients, and a higher bone density. Acute VCFs with no increased uptake or low levels of uptake were found only within the first six days of the trauma. **Conclusions**: BS detects radiologically occult fractures and can differentiate if a radiographically evident VCF is indeed clinically active, guiding possible treatment options. To avoid missing acute VCFs, BS should be performed six days or more after the injury.

## 1. Introduction

Vertebral compression fractures (VCFs) are one of the hallmark fractures of osteoporosis and are prevalent in the elderly population. The radiographic appearance of recent (fresh) VCFs and healed (old) VCFs can be similar, but since in acute and symptomatic VCFs, interventional procedures such as percutaneous vertebroplasty (VP), kyphoplasty (KP), or spine fusion surgery may be indicated [1], further imaging studies are needed to determine the fracture’s age and guide appropriate patient management [2].

Magnetic resonance imaging (MRI) is the imaging method of choice for determining a VCF’s age [3,4]. Acute fractures exhibit a low signal intensity on T1-weighted sequences and a high signal intensity on T2-weighted sequences. The abnormal signal gradually disappears within 2–4 months [5]. 

Bone scintigraphy (BS) using Tc99m-MDP can also be an effective method for determining the age of VCFs and is utilized mainly when MRI is unavailable or contraindicated. 

In BS, an acute VCF presents as an intense horizontal linear tracer uptake in a vertebral body. This pattern usually appears within the first 48 h and decreases in intensity or fades over a period of 6 to 24 months [6]. Blood pool scintigraphy performed early, up to 5 min after injection of Tc-99m MDP, can demonstrate areas of hyperemia, also indicating an acute process [7].

In recent years, several small retrospective studies have compared the appearance of VCFs on MRI and BS with discordant results [8,9,10]. In one study, 30 patients who had undergone both BS and MRI evaluation were reviewed retrospectively with good consistency between the studies [11]. In a study of 30 patients with multilevel vertebral compression fractures, BS was found to better localize the fresh fracture causing clinical symptoms than MRI [12]. The small numbers in these studies and the varying outcome measures make it difficult to conclude whether BS can safely replace MRI as a method for determining a fracture’s age and clinical relevance. 

The aim of this study was to suggest a treatment algorithm utilizing BS rather than MRI as a guide in the decision-making process, by describing the patterns and temporal dynamics of VCFs on BS as related to the fracture type, age, and patient characteristics in a large patient population when MRI is unavailable in the initial evaluation and treatment phase.

## 2. Materials and Methods

This study was conducted retrospectively and was approved by our medical center’s institutional review board (approval number 0038-20-NHR). The need for written informed consent was waived.

### 2.1. Study Population

Included were 200 consecutive patients admitted to our hospital between March 2017 and April 2022 with suspected acute VCFs in the thoracic and lumbar spine demonstrated on CT, corresponding with A1-type fractures according to the AOSpine Thoracolumbar Spine Injury Classification System and having undergone a BS were included [13]. Non-A1 fractures, systemic conditions affecting the skeleton (such as disseminated multiple myeloma, metastatic disease, or endocrine illnesses other than osteoporosis), as well as a technically inappropriate CT or BS were excluded, leaving 190 patients for further analysis.

### 2.2. Data Collection

The demographic and clinical information collected including age, gender, previously diagnosed osteoporosis, previously recorded trauma, and date of the trauma were retrieved from our hospital’s electronic health records (EHRs).

CT of the spine was acquired using one of two CT scanners (Philips Ingenuity 128 and Brilliance 64, Cleveland, OH, USA). The CT parameters were as follows: tube voltage, 120 kV; tube current, 120–190 mA; slice thickness, 2 mm; detector collimation, 64 × 0.625 mm or 128 × 0.625 mm; gantry rotation time, 1; and pitch, 0.8. All CT data were reconstructed to a slice thickness of 2 mm, with a 512 × 512 matrix using a soft-tissue kernel. No oral or intravenous (IV) contrast media were administered. Sagittal and coronal reformatted images with a slice thickness of 2 mm were routinely created. In 161 patients, the CT included the thoracic and lumbar spine, and in 29 patients, only the lumbar spine. A total of 2966 vertebrae were scanned in 190 patients.

The CT images were reviewed by a radiologist and a spine surgeon for the presence of vertebral fractures at the time of admission. A spine surgeon retrospectively reviewed the CT images for the purpose of this study, defined the fractures as acute or chronic, and classified them using the AOSpine Thoracolumbar Spine Injury Classification System (AO type). Only A-type VCFs (compression injury) were included in the study. Signs of degenerative disc changes, including end plate sclerosis, irregularity, and osteophytes were noted separately.

**Figure 1:**
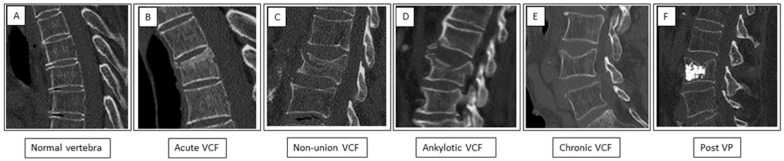
Patterns of vertebrae appearance on sagittal spine CT. (**A**) Normal vertebra—retained height and continuous cortex. (**B**) Acute VCF showing a “step defect” in the anterior border and a “zone of impaction” caused by impaction of the trabeculae. (**C**) Non-union fracture—a non-healed fracture with an intervertebral cleft (white arrow). (**D**) Ankylotic fracture—a transverse fracture below an ankylotic spine segment. (**E**) Chronic fracture showing loss of height and smooth cortical borders. (**F**) State after percutaneous vertebroplasty (Post VP)—chronic fracture with loss of height and hyperdense cement in the vertebral body.

A description of the CT pattern was documented for each vertebra according to the following (Figure 1): (A) normal vertebra (no fracture); (B) acute VCF displaying CT signs of loss of height or anterior wedge deformity with endplate irregularity, cortical incontinuity, step defects, increased density zone of impaction, and soft-tissue edema or hematoma surrounding the vertebral body; (C) non-union of a VCF displaying a non-healed fracture with an intravertebral cleft, also known as Kümmell’s disease; (D) ankylotic VCF displaying a transverse fracture below an ankylotic spine segment; (E) chronic VCF displaying loss of height or anterior wedge deformity with smooth cortical borders; and (F) following percutaneous vertebroplasty (Post VP) displaying cement within the vertebral body. 

The bone density was estimated for each patient using CT. Hounsfield units (HUs) were measured by placing a region of interest (ROI) in the intramedullary area of a lumbar vertebral body, preferably L3, unless fractured. Values measured below 118 HUs were consistent with osteopenia (T-score of −1.0 to −2.5). Values below 93 HUs were consistent with osteoporosis (T-score of −2.5 or less) [14]. 

BS was performed using one of two gamma cameras (Infinia Hawkeye and Optima 640, GE Medical Systems, Milwaukee, WI, USA) with a large field-of-view, dual-head, single-photon emission computed tomography (SPECT) system fitted with low-energy, high-resolution collimators. Whole-body planar acquisition was carried out using the continuous method in a 256 × 1024 matrix. SPECT images were obtained in a 128 × 128 matrix with a 20% window centered at 140 keV and were reconstructed with ordered subsets expectation maximization (OSEM), using 2 iterations. Early planar imaging of the spine and pelvis was performed 5–10 min after IV administration of 20–25 mCi (~740 MBq) of Tc99m-MDP (Jubilant Draxlmage Inc, Canada) and late whole-body planar images and SPECT of the thoracic and lumbar spine were performed 2–4 h after injection. Early BS of the spine was performed in 185 patients (97%). All patients underwent late planar whole-body imaging and SPECT of the thoracic and lumbar spine with 2 fields of view (FOV). A total of 3230 vertebrae were scanned. All BS was reviewed by a board-certified nuclear medicine physician and findings on planar and SPECT images were documented for each vertebra. For early images, the presence or absence of increased blood pool was recorded. For late images, a 3-point visual score of the uptake pattern was documented according to the following: low intensity uptake, slightly above that of adjacent normal vertebra; intermediate intensity uptake, clearly above that of adjacent normal vertebra but below uptake in the sacroiliac joint (SIJ) or anterior superior iliac spine (ASIS); and high intensity uptake, similar or above uptake in the SIJ or ASIS.

Evaluation of the BS was performed unblinded to the findings on CT, which were used to accurately define the location of fractures and to distinguish between uptake caused by other etiologies such as osteophytes and degenerative changes. If the increased uptake was deemed to be the result of degenerative changes seen on the CT (rather than a fracture of the vertebral body), the uptake was excluded from the study.

### 2.3. Statistical Analysis

Data analysis was conducted using SPSS software, version 27.0 for Windows (IBM, New York, NY, USA). For normally distributed data, the results were presented as mean ± standard deviation and were analyzed using an unpaired Student’s *t*-test. Categorical data were expressed as percentages to illustrate their distribution, and a *p*-value of less than 0.05 was considered to indicate statistical significance. To explore the relationship between bone density, sex, and age, Pearson’s correlation test was employed. This test allowed for the assessment of the strength and direction of the linear relationship between these two continuous variables. Additionally, multivariate logistic regression analysis was performed to identify factors that could influence the likelihood of VCF detection. The variables included in this analysis were age, gender, the time elapsed between the trauma and scanning, and bone density. By incorporating these factors, the regression model aimed to determine the individual and combined effects on the odds of VCF detection provide a comprehensive understanding of the potential predictors in this clinical context.

## 3. Results

### 3.1. Patient Population

Two hundred consecutive patients were initially included. Ten patients were excluded from further analysis due to technically inappropriate or missing imaging studies or to etiology of fractures secondary to malignancy or multiple myeloma. A total of 2966 vertebrae were scanned in 190 patients.

The demographic data of the final study population of 190 patients are detailed in Table 1. Forty-three patients had documented osteoporosis on their electronic health records. In 108 patients (57%), a trauma event with a specific date was recorded; 19 patients (10%) had a known history of recent trauma within the previous two weeks but could not recall the exact date; and 63 patients (33%) had no history of a trauma event. In patients with a recorded trauma date, the mean time between the trauma and hospitalization was 6.1 days, and the mean time between the injury and BS was 8.3 days. All patients underwent CT within the first 48 h of admission. BS was acquired 48 h after the CT, except in four cases in which the study was performed on the same day or a day before the CT. The mean time interval between CT and BS was 3.1 days (see Table 1). An MRI study was not obtained in any of the patients.

### 3.2. CT Findings

**Figure 2:**
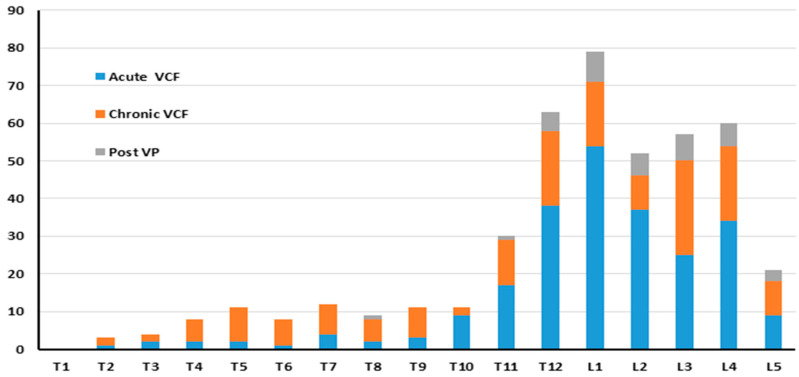
Distribution of VCFs according to type and location on CT.

In CT, 84 patients (44.2%) had a single acute VCF, 48 patients (25.2%) had 2 VCFs, 26 patients (13.7%) had 3 acute VCFs, and 32 patients (16.8%) had more than 3 acute VCFs, with the highest number being 11 acute VCFs in a single patient. VCFs were observed in 439 vertebrae on CT, including 240 which had radiologic patterns of acute VCFs (54.7%), 162 chronic VCFs (36.9%), and 37 post VP VCFs (8.4%). Most of the VCFs were located between T11 and L4, with L1 being the most common location (Figure 2). Among the acute VCFs, 49 were non-union and 16 were fractures below an ankylotic segment.

### 3.3. Bone Density

The CT-measured mean bone density was 61.2 ± 32 HU, with a range of −21–164 HUs. Values above 118 HUs, considered normal, were found in 10 patients (5.2%). Values of 93 to 118 HUs, consistent with osteopenia, were found in 17 patients (8.9%), and values below 93 HUs, consistent with osteoporosis, were found in 163 patients (85.7%). The mean bone density in women was significantly lower compared to men (55.6 ± 31 HUs compared to 73.3 ± 31 HUs, *p* < 0.001) and lower values were found with increasing age of the patients in both men and women (r = −0.368, *p* < 0.001).

### 3.4. BS Findings

**Figure 3:**
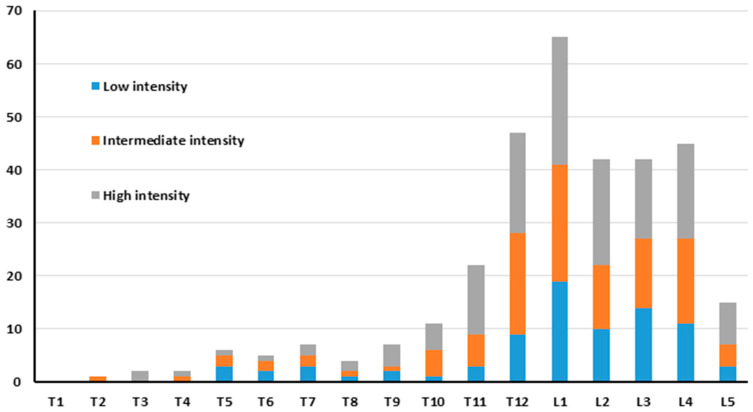
Distribution of VCFs according to uptake intensity and location on bone scintigraphy.

In BS, increased uptake, consistent with a vertebral fracture, was observed in 323 vertebrae. The uptake intensity was low in 81 (25%) vertebrae, intermediate in 107 (33%), and high in 135 (42%) vertebrae. Most of the vertebrae with an abnormal uptake were seen in the thoraco-lumbar region (Figure 3). 

In early BS, increased blood pool was observed in 193 vertebrae (60%). In late BS, vertebrae showing hyperemia in early studies had higher uptake intensities; a total of 121 vertebrae (63%) had a high intensity uptake, 60 (31%) had intermediate, and 12 (6%) had low-level uptake. Increased late tracer uptake without hyperemia was found in 130 vertebrae (40%), with lower uptake intensities; a total of 14 vertebrae had high-intensity uptake (11%), 47 had intermediate (36%), and 69 had low-intensity uptake (53%). Hyperemia was more prevalent in the lower thoracic and lumbar spine (T11 to L5, 63%) compared to the high and mid-thoracic spine (T1 to T10, 42%). 

**Figure 4:**
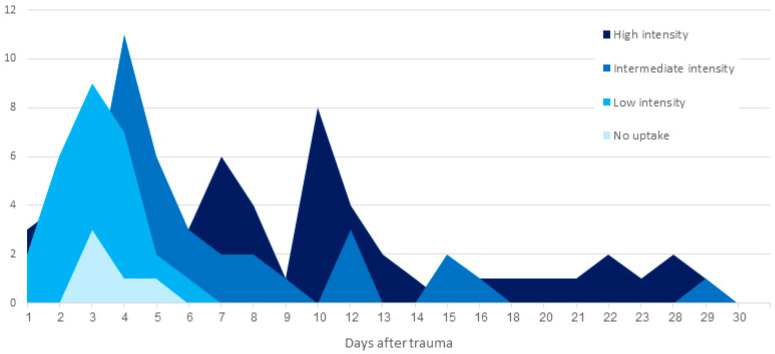
Radiotracer uptake intensity according to time after trauma.

In a subgroup of 108 patients with a known date of the traumatic event and 132 acute VCFs according to CT, BS uptake intensity was compared to the time period elapsed since the trauma. Acute VCFs with no increased uptake were found up to five days after trauma, and VCFs with low-intensity uptake were seen up to six days after trauma. Acute VCFs with intermediate- and high-intensity uptake were seen from the first day after trauma up to 38 and 62 days, respectively (Figure 4). 

Increased uptake in ribs, sacrum, and pelvis, consistent with acute fractures, were seen in the majority of the patients (160, 84%) in BS. Single-rib fractures were observed in 28 patients, multiple-rib fractures in 63 patients, sacral fractures in 55, and pelvic fractures in 14 patients.

### 3.5. Comparison of CT and BS Findings

**Figure 5:**
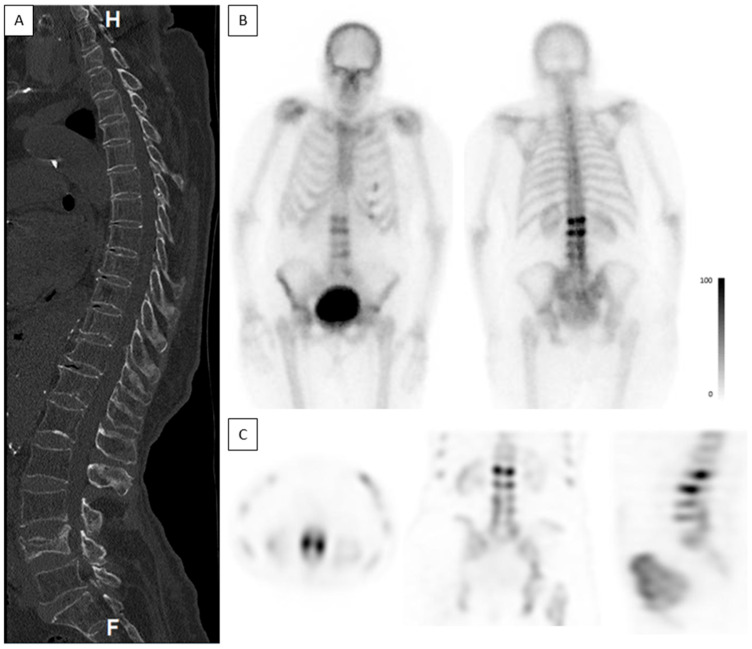
Occult fractures. A 78-year-old female complaining of back pain, without known trauma. CT was acquired on the day of admission and bone scintigraphy was performed 2 days after the CT. (**A**)—Sagittal spine CT shows a non-union fracture in L4. No other fractures are demonstrated. Bone density measured in L3 was 7 HUs, consistent with severe osteoporosis. (**B**)—Planar anterior and posterior bone scintigraphy shows high-intensity uptake in L2 and L3, suggestive of acute fractures. (**C**)—Axial, coronal, and sagittal SPECT show high-intensity uptake in L2 and L3.

**Figure 6:**
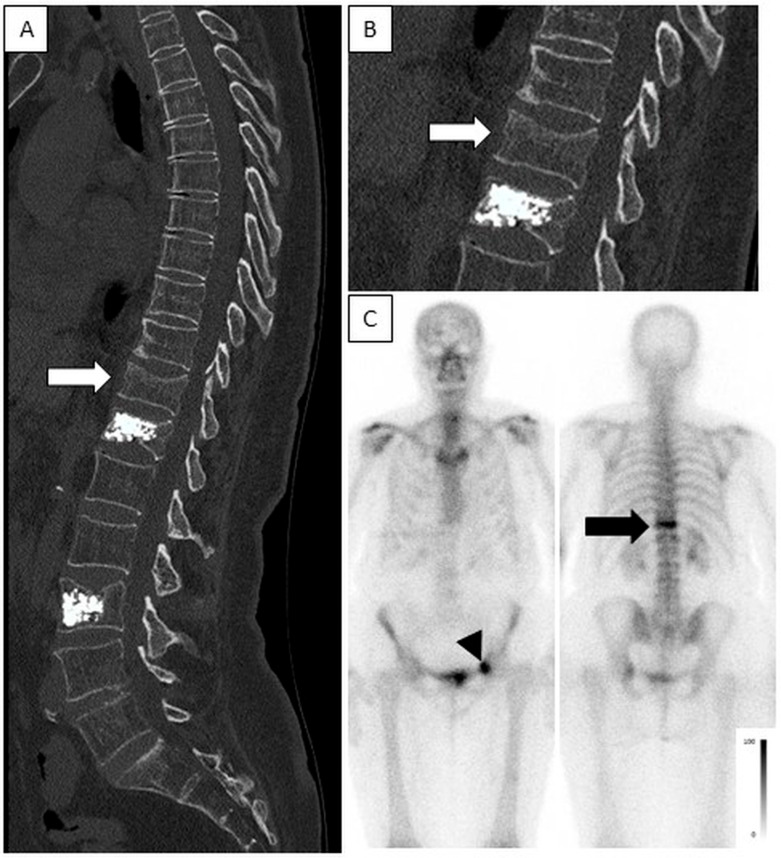
High-intensity uptake in an acute fracture, no uptake in chronic fractures post-vertebroplasty. A 77-year-old female after trauma. CT was acquired on the day of injury and bone scintigraphy was performed 3 days after the injury. (**A**,**B**)—Sagittal spine CT (enlarged in B) show an acute fracture in T11 (white arrow) and chronic fractures after vertebroplasty in T12 and L3. (**C**)—Planar anterior and posterior bone scintigraphy show high-intensity uptake in T11 (black arrow), indicating an acute fracture and no increased uptake in T12 and L3, consistent with chronic fractures. Note also, high-intensity uptake is seen in the anterior aspect of the left acetabulum (black arrow head), consistent with an acute fracture.

**Figure 7:**
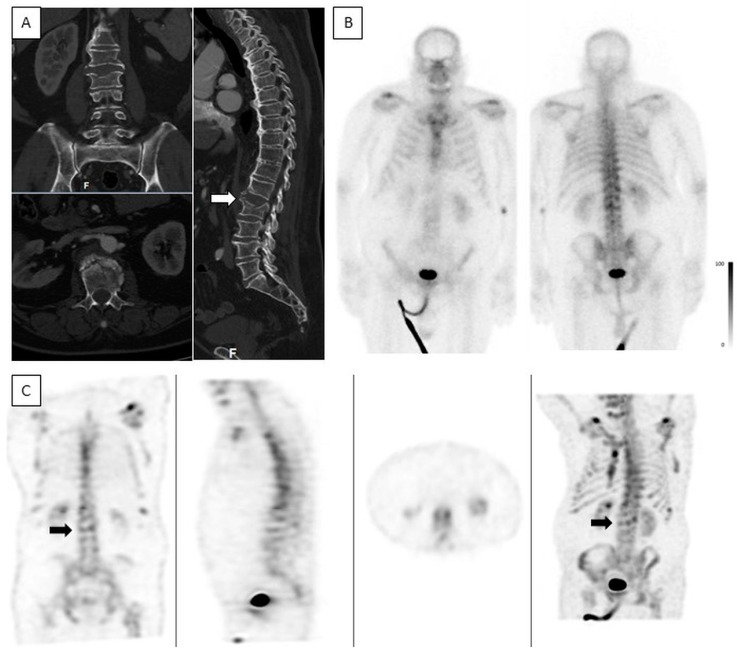
Low-intensity uptake in an acute ankylotic VCF. A 72-year-old male. CT was acquired on the day of injury and bone scintigraphy was performed 3 days after the injury. (**A**)—Coronal, axial, and sagittal spine CT show an acute ankylotic fracture in L2 and a fracture involving an osteophyte in L1 (white arrow). (**B**)—Planar anterior and posterior bone scintigraphy do not show any increased uptake in L2. (**C**)—Coronal, sagittal, axial, and MIP SPECT show only low-intensity uptake in L2 (black arrow).

The imaging patterns of CT and BS were compared in 2966 vertebrae. Table 2 and Figure 5, Figure 6 and Figure 7 demonstrate the different combined patterns of CT and BS.

**Normal vertebrae**—In the group of vertebrae with a normal appearance on CT, most had no increased uptake on BS (2504/2527, 99%). Increased uptake was seen in 23 (0.9%) vertebrae that appeared normal on CT, consistent with the diagnosis of an acute radiologically occult fracture (Figure 5). This pattern combination was observed in 17 patients. Multivariate logistic regression analysis of these patients indicated that an increase in age (OR = 0.913, 95% CI [0.840, 0.993], *p* = 0.035) and a decrease in bone density (OR = 0.969, 95% CI [0.939, 1.000], *p* = 0.052) were associated with a decrease in the odds of detection of a VCF on the CT. Notably, these associations remained statistically significant, even after controlling for gender and the time elapsed between the trauma and scanning. Gender and time elapsed from trauma had no significant impact on the likelihood of detection of an occult fracture.

**Acute VCFs**—Most vertebrae with acute fractures on CT had an increased uptake on BS (233/240, 97%), with 79 and 111 showing intermediate- and high-intensity uptake, respectively (Figure 6), and 43 showing low-intensity uptake (Figure 7). In seven acute VCFs, no increased uptake was seen on BS. The pattern combination of acute VCF with no increase or low-intensity uptake was observed in 40 patients. The clinical characteristics of these patients were compared to the group with intermediate- or high-intensity uptake. In univariate analyses, the time elapsed between the trauma and BS was significantly shorter in these patients (4.62 ± 6.18 vs. 9.68 ± 11.48 days, *p* = 0.026) and male gender was more prevalent (31.7% vs. 16.2%; *p* = 0.015). Bone density was higher, but not statistically significant (69.90 ± 34.92 vs. 58.87 ± 30.98, *p* = 0.053). Multivariate logistic regression analysis also found a significant difference in the time span elapsed from the traumatic event to BS, with a shorter time period in the low-uptake group (OR = 0.908, 95% CI [0.822, 1.003], *p* = 0.057). No association was found with patient’s age. 

Hyperemia was seen in the early BS images in 157 acute VCFs (65%). In 83 acute VCFs (35%), no hyperemia was observed. 

In non-union VCFs, intermediate- and high-intensity uptakes were more prevalent (41 vertebrae, 84%), with few cases showing no increase in uptake or low-intensity uptake (8 vertebrae, 16%). In ankylotic VCFs, intermediate- and high-intensity uptakes were found in 12 vertebrae (63%) and low-intensity or no increase in uptake were observed in 7 vertebrae (37%). Univariate or multivariate logistic regression analysis were not possible due to the small number of cases.

**Chronic VCFs**—Among 162 vertebrae with chronic fractures according to CT, 117 did not display increased uptake (72%). Low-intensity uptake was observed in 23 vertebrae (14%), and high- and intermediate-intensity uptake was seen in 15 and 7 vertebrae (9% and 4%), respectively. The pattern combination of chronic VCFs with intermediate- or high-intensity uptake was observed in 20 patients, suggestive of an unstable or “active” fracture. Multivariate analysis of these patients’ clinical characteristics showed a significantly lower bone density (43.55 vs. 63.26 HU, *p* = 0.009) and a trend towards female gender that was not statistically significant (*p* = 0.092).

**Post VP**—Most vertebrae in this group showed no increase (15, 40%) or a low-intensity uptake (12, 32%), and few demonstrated intermediate- and high-intensity uptake (7, 19% and 3, 8%, respectively).

## 4. Discussion

The present study identified and characterized 2966 vertebrae in 190 patients with 439 VCFs. The study population was mostly elderly and female. Only 5% of the patients had normal bone density, estimated using spine CT, and over 85% had osteoporosis according to these measurements. Most patients (55.8%) had more than one VCF, with 16.8% of them having three or more VCFs. In some cases, a combination of acute and chronic fractures was found. Most of the fractures were located in the thoraco-lumbar area.

In most patients, the CT results were retrospectively sufficient to determine whether a vertebra was normal or had either acute or chronic VCFs, but in 37 (19.5%) patients, BS added important information related to the fracture that could influence patient management, as radiologically occult fresh fractures were found in 23 normal-appearing vertebrae and in 22 vertebrae with the radiographic appearance of chronic fractures. These occult fractures were amenable to surgical intervention, which could potentially improve pain relief and facilitate patient mobilization [1].

The phenomenon of radiologically occult fractures, also known as “VCFs without radiologic collapse”, has been described in several studies. Pham et al. described 16 osteoporotic subjects with acute, severe back pain, but no evidence of VCF on a lateral spine radiograph. MRI or BS showed findings consistent with acute fractures in an anatomic distribution, correlating with the clinical pain. The subjects were followed prospectively with radiographic changes, consistent with VCFs developing in 80% of subjects by the end of the study [15]. Additional studies and reviews have described this phenomenon on radiographs [16,17,18,19,20] but not on CT. Our study has demonstrated that occult fractures may also be present and missed on diagnostic, high-resolution CT or may be present in a vertebra with imaging characteristics of a chronic fracture. In multivariate logistic regression analysis, occult fractures in normal-appearing vertebrae on CT were associated with an increase in patient age and a decrease in bone density. This could be explained by the lower visibility of cortical disruption and the impaction of the trabeculae in osteoporotic vertebrae, which have thinner and less dense trabeculae and corteces. The patient’s gender and the time elapsed between the traumatic event and scanning had no significant impact on the likelihood of detection of an occult fracture on BS. 

In our study group, 40 patients had acute VCFs with no increase or low-intensity uptake on BS. These were observed up to 6 days after injury. According to the known literature, increased radiotracer uptake appears in vertebral fractures on BS during the osteoblastic activity phase, signifying the initiation of the healing process, usually during the first hours after injury. In many cases, fractures can be seen on BS much earlier than on radiographs or even CT, mainly in rib and sacral fractures [7]. In the seminal study by Matin et al. from 1979, the appearance of various fractures on BS over time was reported in 204 patients aged between 17 and 88 years, with a third of patients being over 65 years [21]. In their study, 80% of all fractures showed an abnormal uptake by 24 h, and 95% by 72 h after injury. Only in two patients, both over the age of 65 years, there was no increased uptake on BS in a known acute fracture by 72 h. While this study suggests that in the elderly population, scintigraphic abnormalities may appear later than in younger patients, it did not focus on vertebral fractures and had only a limited number of elderly patients. In our study, a higher percentage of patients had no uptake or low-grade uptake in acute VCFs for a longer period of time, suggesting that a longer lag period between the injury and BS is needed to prevent a false-negative diagnosis.

Another study by Spitz et al. from 1992 investigated the appearance of fractures on BS in 480 patients, including 123 fractures in the thoracic and lumbar spine and 357 fractures in other bones, including the radius, scaphoid, femoral neck, pelvis, and shaft of long bones [22]. In 18 patients, repeated scanning was conducted during the initial 24 h after injury. All acute fractures showed increasing accumulation of Tc-99m MDP at the fracture site in those 24 h and within 2 weeks after injury, but the magnitude of the increase was markedly different in different bones. Spine fractures showed the latest and slowest accumulation of Tc-99m MDP, with some fractures appearing only 10 to 12 days after the injury. The authors suggested that these findings are related to the amount of callus formation found in fractures adjacent to joints compared to vertebral fractures, and that a similar behavior can be seen in skull fractures that show minimal uptake on BS and almost no callus formation on radiographs. Multiple-regression analysis of their data did not find any correlation between the time of appearance of the fracture and the patient’s age and gender, in contrast to Matin et al.’s study. The authors concluded that the rate of accumulation of Tc-99m MDP is only dependent on the fracture site, with slower accumulation in the spine and the shafts of long bones. 

The appearance of vertebral fractures over time on BS has not been investigated in more recent years. Our results show that the time of appearance of increased tracer uptake is not dependent only on age, as suggested by Matin et al., or only on the timing of BS and the fracture location, as suggested by Spitz et al., but is influenced by multiple factors, including the injury to BS lag period, the patient’s gender, and bone density. In the sub-group of ankylotic fractures with no uptake or low-grade uptake, we also observed a higher prevalence of males, but this was not statistically significant. Since diffuse idiopathic skeletal hyperostosis (DISH) occurs more in males [23,24], this may explain their higher prevalence in this group. A further study of this population is needed to elucidate the reasons for this phenomenon.

**Figure 8:**
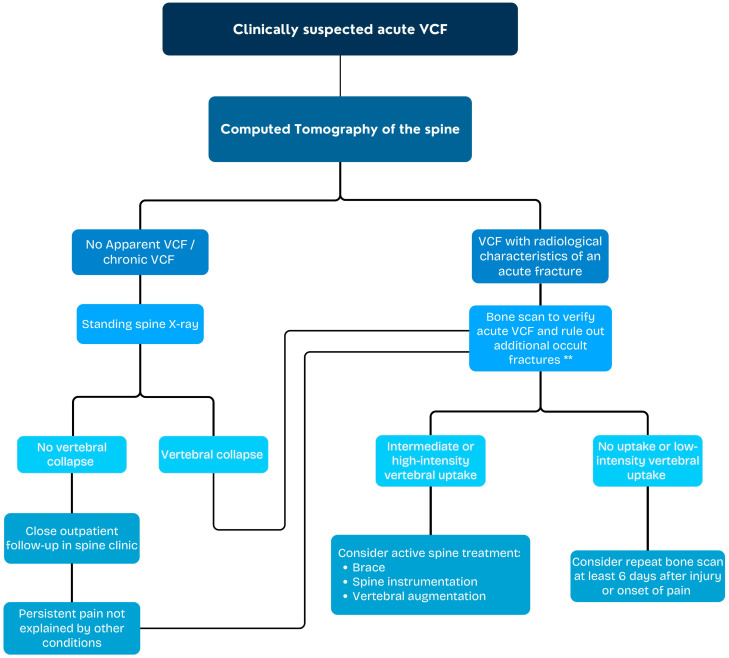
VCF diagnostic flow-chart. When MRI is unavailable or contraindicated. ** Bone scan should be obtained at least 48 h after injury or onset of pain.

According to orthopedic guidelines, MRI is considered the gold-standard method for determining the acuity of a VCF, while the combination of CT and BS is reserved for claustrophobic patients or for MRI incompatibility [3,4,25]. Only one guideline refers to MRI and BS equally as “advanced imaging” that can reliably confirm the presence and location of acute VCFs that may be amenable to treatment [26]. Our study provides additional evidence demonstrating that in clinical scenarios when MRI is unavailable in the acute setting, a combination of spine CT and BS can be used for establishing the age of vertebral fractures and to detect additional occult fractures, supporting clinical decision-making. This is visualized in Figure 8, in which a diagnostic flow-chart algorithm is provided.

While BS is considered a sensitive method for detecting acute fractures and for determining a fracture’s age [6,27], several studies report discordance between the results of MRI and BS concerning the establishment of the age of VCFs. A study by Masala et al. found that MRI and BS are concordant for fractures up to 4 months old [8]. Kim et al. found an overall concordance of only 55% between the methods. The concordance for single-level VCFs was very high (96%), with a significant drop in concordance in two-level (50%) and three-level fractures (only 36%) [9]. In a study by Dafydd et al. from 2014, the overall concordance was of 63%, with almost twice as many acute or subacute fractures found on BS [10]. These studies suggest that acute fractures appear on BS for a longer period of time than on MRI. In multilevel fractures, the different VCFs probably have different ages, with older fractures not apparent on MRI but still seen on BS. Therefore, it can be assumed that the information regarding the temporal dynamics is not equivalent in BS and MRI. Despite the discordance between the methods, BS was found to be a useful method for locating the painful vertebra before deciding on surgical treatment [28,29,30,31]. Most of these studies had a small number of up to 44 patients, none of them used an early blood pool phase, and SPECT/CT was used only in a single study. To the best of our knowledge, this is the first study comparing imaging patterns of VCFs in BS and CT at varying time points, using modern, state-of-the-art equipment. 

In our study, an early blood pool phase scan was acquired in 97% of the patients, and all studies included SPECT of the thoracic and lumbar spine. The majority of acute VCFs, 65%, showed hyperemia. The fact that 83/240 (35%) of these fractures demonstrated no hyperemia may be explained by relatively older aged fractures. Early blood pool images are characterized by low-resolution and unfavorable target-to-background ratios and, in the thoracic spine, vertebral hyperemia is often obscured by uptake in the mediastinum. Therefore, it might be concluded that although hyperemia can be considered a definite sign of acute fracture, its sensitivity is low and its absence should not rule out an acute VCF.

SPECT studies are known to increase the sensitivity and specificity of BS [32]. In the current study, SPECT images were very helpful in detecting low-intensity uptakes that would have been missed using only planar images, as shown in Figure 7. They were also paramount for determining the exact location of the fractures and were easier to compare to the recently performed spine CT. The combined viewing of the SPECT and CT images was helpful in differentiating uptakes caused by degenerative changes vs. true fractures. If spine CT is not acquired prior to the bone scintigraphy in patients with suspected acute VCF, SPECT/CT should be performed when available.

The imaging patterns observed in the current study may be extrapolated in the future, after additional research, to other bone imaging scintigraphy methods such as F18-fluoride positron emission tomography (PET) or PET/MR. 

Our study was limited by using retrospective data. More definite data may have been gained by repeating BS at decided time points during the first week after injury, but this was not clinically feasible. Estimation of osteopenia and osteoporosis in our patient population was performed using an opportunistic method applied to the previously acquired spine CT. Although this method was investigated and found to accurately represent mineral bone density [14], using dual x-ray absorptiometry would have been more precise.

### Main Findings in This Study

In 19.5% of our patients, a BS added important information related to the fracture that could influence patient management.

A fresh VCF may be present in a vertebra with imaging characteristics of a chronic fracture.

A fresh VCF may be present with CT characteristics of a normal vertebra.

Although hyperemia can be considered a definite sign of an acute fracture, its sensitivity is low and its absence should not rule out an acute VCF

BS is equivalent to MRI as a corroborating modality in determining the age of VCFs found on radiographs or CT.

## 5. Conclusions

Bone scans, in the setting of vertebral compression fractures in the elderly population, add valuable information on the fracture’s age, can detect occult fractures, and can subsequently affect patient management. The ideal timing of the scan should be considered as in some cases, no increased uptake or only low-intensity uptake was demonstrated in acute fractures up to six days after trauma. 

## Figures and Tables

**Table 1:** Patient characteristics.

		*n*	%	Mean	SD	Minimum	Maximum
Gender	male	60	31.6				
	female	130	68.4				
Age		190		75	11.2	36	98
Time between CT and BS		190		3.1	4.8	−1	33
Time between the trauma and hospitalization		108		6.1	10.7	0	61
Time between the trauma and BS		108		8.3	10.5	1	62

**Table 2:** Prevalence of combined CT and BS patterns (*n* = 2966).

CT Pattern	BS Uptake Intensity	Number	%
Normal	No increased uptake	2504	84.4
Normal	Low	3	0.1
Normal	Intermediate	6	0.2
Normal	High	14	0.5
Acute VCF	No increased uptake	7	0.2
Acute VCF	Low	43	1.4
Acute VCF	Intermediate	79	2.7
Acute VCF	High	111	3.7
Chronic VCF	No increased uptake	117	3.9
Chronic VCF	Low	23	0.8
Chronic VCF	Intermediate	15	0.5
Chronic VCF	High	7	0.2
Post VP	No increased uptake	15	0.5
Post VP	Low	12	0.4
Post VP	Intermediate	7	0.2
Post VP	High	3	0.1

## Data Availability

Data used for this study may be obtained, on request, from the corresponding author.

## References

[1] Kondo K.L. (2008). Osteoporotic vertebral compression fractures and vertebral augmentation. Semin. Intervent. Radiol..

[2] Rahamimov N., Arnon-Sheleg E. (2021). Identifying Multi-Level Vertebral Compression Fractures Following a Convulsive Seizure. Isr. Med. Assoc. J..

[3] McConnell C.T., Wippold F.J., Ray C.E., Weissman B.N., Angevine P.D., Fries I.B., Holly L.T., Kapoor B.S., Lorenz J.M., Luchs J.S. (2014). ACR Appropriateness Criteria Management of Vertebral Compression Fractures. J. Am. Coll. Radiol..

[4] Hirsch J.A., Beall D.P., Chambers M.R., Andreshak T.G., Brook A.L., Bruel B.M., Deen H.G., Gerszten P.C., Kreiner D.S., Sansur C.A. (2018). Management of vertebral fragility fractures: A clinical care pathway developed by a multispecialty panel using the RAND/UCLA Appropriateness Method. Spine J..

[5] Lin H.-H., Chou P.-H., Wang S.-T., Yu J.-K., Chang M.-C., Liu C.-L. (2015). Determination of the painful level in osteoporotic vertebral fractures—Retrospective comparison between plain film, bone scan, and magnetic resonance imaging. J. Chin. Med. Assoc..

[6] Vali R., Bajno L., Kousha M., Martin C. (2016). The Potential Role of Radionuclide Imaging in Osteoporotic Vertebral Fracture and Sacral Fracture. Spine Res..

[7] Ziessman H.A., O’Malley J.P., Thrall J.H. (2006). Nuclear Medicine: The Requisites in Radiology.

[8] Masala S., Schillaci O., Massari F., Danieli R., Ursone A., Fiori R., Simonetti G. (2005). MRI and bone scan imaging in the preoperative evaluation of painful vertebral fractures treated with vertebroplasty and kyphoplasty. In Vivo.

[9] Kim J.-H., Kim J.-I., Jang B.-H., Seo J.-G., Kim J.-H. (2010). The Comparison of Bone Scan and MRI in Osteoporotic Compression Fractures. Asian Spine J..

[10] Ap Dafydd D., Salem S., Zerizer I., Yan Mok W., Gishen P., Patel M.C., Patel N.H., Al-Nahhas A., Dunn J., Win Z. (2014). The value of combined assessment of vertebral fractures with 99mTc MDP scintigraphy and MRI in selecting and planning percutaneous vertebroplasty. Nucl. Med. Commun..

[11] Zhao Y., Zhang T., Tang P. (2022). Diagnostic value of SPECT/CT bone imaging in fresh osteoporotic vertebral compression fractures. Hell. J. Nucl. Med..

[12] Okazaki T., Nakagawa H., Yagi K., Hayase H., Nagahiro S., Saito K. (2017). Bone scintigraphy for the diagnosis of the responsible level of osteoporotic vertebral compression fractures in percutaneous balloon kyphoplasty. Clin. Neurol. Neurosurg..

[13] Vaccaro A.R., Oner C., Kepler C.K., Dvorak M., Schnake K., Bellabarba C., Reinhold M., Aarabi B., Kandziora F., Chapman J. (2013). AOSpine thoracolumbar spine injury classification system: Fracture description, neurological status, and key modifiers. Spine.

[14] Schreiber J.J., Anderson P.A., Rosas H.G., Buchholz A.L., Au A.G. (2011). Hounsfield Units for Assessing Bone Mineral Density and Strength: A Tool for Osteoporosis Management. J. Bone Jt. Surg..

[15] Pham T., Azulay-Parrado J., Champsaur P., Chagnaud C., Legré V., Lafforgue P. (2005). “Occult” Osteoporotic Vertebral Fractures. Spine.

[16] Yang X., Mi S., Mahadevia A.A., Lin X., Shi W., Liu A., Li L., Wu Z., Murphy K. (2008). Pain reduction in osteoporotic patients with vertebral pain without measurable compression. Neuroradiology.

[17] McKiernan F.E. (2009). The broadening spectrum of osteoporotic vertebral fracture. Skeletal Radiol..

[18] Kim Y.J., Chae S.U., Kim G.D., Park K.H., Lee Y.S., Lee H.Y. (2013). Radiographic Detection of Osteoporotic Vertebral Fracture without Collapse. J. Bone Metab..

[19] Wáng Y.X.J., Che-Nordin N. (2019). Some radiographically ‘occult’ osteoporotic vertebral fractures can be evidential if we look carefully. Quant. Imaging Med. Surg..

[20] Du M.-M., Che-Nordin N., Ye P.-P., Qiu S.-W., Yan Z.-H., Wang Y.X.J. (2020). Underreporting characteristics of osteoporotic vertebral fracture in back pain clinic patients of a tertiary hospital in China. J. Orthop. Transl..

[21] Matin P. (1979). The appearance of bone scans following fractures, including immediate and long-term studies. J. Nucl. Med..

[22] Spitz J., Lauer I., Tittel K., Wiegand H. (1993). Scintimetric evaluation of remodeling after bone fractures in man. J. Nucl. Med..

[23] Fournier D.E., Leung A.E., Battié M.C., Séguin C.A. (2024). Prevalence of diffuse idiopathic skeletal hyperostosis (DISH) and early-phase DISH across the lifespan of an American population. Rheumatology.

[24] Ahmed O., Ramachandran K., Patel Y., Dhanapaul S., Meena J., Shetty A.P., Bhari Thippeswamy P., Kanna R.M., Rajasekaran S. (2022). Diffuse Idiopathic Skeletal Hyperostosis Prevalence, Characteristics, and Associated Comorbidities: A Cross-Sectional Study of 1815 Whole Spine CT Scans. Glob. Spine J..

[25] Bravo A.E., Brasuell J.E., Favre A.W., Koenig B.M., Khan A.A., Beall D.P. (2020). Treating Vertebral Compression Fractures: Establishing the Appropriate Diagnosis, Preoperative Considerations, Treatment Techniques, Postoperative Follow-Up and General Guidelines for the Treatment of Patients With Symptomatic Vertebral Compression Fractu. Tech. Vasc. Interv. Radiol..

[26] Barr J.D., Jensen M.E., Hirsch J.A., McGraw J.K., Barr R.M., Brook A.L., Meyers P.M., Munk P.L., Murphy K.J., O’Toole J.E. (2014). Position Statement on Percutaneous Vertebral Augmentation: A Consensus Statement Developed by the Society of Interventional Radiology (SIR), American Association of Neurological Surgeons (AANS) and the Congress of Neurological Surgeons (CNS), American Col. J. Vasc. Interv. Radiol..

[27] Martin J.G., Goldman D.T., Dabrowiecki A.M., Newsome J., Bercu Z.L., Gilliland C. (2020). Additional Magnetic Resonance or Nuclear Scintigraphy Imaging Influences Approach to Vertebral Augmentation. Spine.

[28] Tang Z., Lei Z., Yang H., Chen K. (2012). Value of Bone Scan Imaging in Determining Painful Vertebrae of Osteoporotic Vertebral Compression Fractures Patients With Contraindications to MRI. Orthop. Surg..

[29] Solá M., Pérez R., Cuadras P., Díaz R., Holgado S., Puyalto P., Iborra M., Fraile M. (2011). Value of bone SPECT-CT to predict chronic pain relief after percutaneous vertebroplasty in vertebral fractures. Spine J..

[30] Jordan E., Choe D., Miller T., Chamarthy M., Brook A., Freeman L.M. (2010). Utility of Bone Scintigraphy to Determine the Appropriate Vertebral Augmentation Levels. Clin. Nucl. Med..

[31] Maynard A.S., Jensen M.E., Schweickert P.A., Marx W.F., Short J.G., Kallmes D.F. (2000). Value of bone scan imaging in predicting pain relief from percutaneous vertebroplasty in osteoporotic vertebral fractures. AJNR. Am. J. Neuroradiol..

[32] Savelli G., Maffioli L., Maccauro M., De Deckere E., Bombardieri E. (2001). Bone scintigraphy and the added value of SPECT (single photon emission tomography) in detecting skeletal lesions. Q. J. Nucl. Med..

